# Happy Software: An interactive program based on an emotion management model for assertive conflict resolution

**DOI:** 10.3389/fpsyg.2022.935726

**Published:** 2023-01-13

**Authors:** Gemma Filella, Agnès Ros-Morente

**Affiliations:** Department of Pedagogy, University of Lleida, Lleida, Spain

**Keywords:** emotions, conflicts, resolution, management, gamification

## Abstract

Emotions are key to conflict resolution: to resolve conflict situations assertively, we must be able to manage the emotions that cause them. After a detailed analysis of the relevant theoretical framework, this paper presents a theoretical emotion management model aimed at assertive conflict resolution. The model, which is described step by step, has been transformed into an interactive program for students, implemented, and assessed in a population aged 8–16 years. The model is divided into four steps or phases. The first is emotional awareness, which consists of understanding and legitimating emotions; the second focuses on reducing emotional intensity and impulsivity; the third encompasses the use of different emotion regulation strategies; and the fourth and final step is assertive communication. Training in this process results in better emotion management, which eventually leads to greater wellbeing and a more positive assessment of new conflicts and aids in the assertive resolution thereof. The final section of the paper summarizes the most important evidence and outcomes of the use of the Happy software so far.

## Introduction

The generation of conflicts is an intrinsic part of human society. A conflict can be defined as a mutual disagreement or hostility between individuals or groups. Conflicts among peers threaten peer relationships and the resources and benefits they offer (Laursen and Adams, 2018). The causes of conflicts are varied—differing needs, interests, identities, values, or opinions—which, in turn, impact our relationships with others (Rozenblum, 2007; Jeong, 2008).

How positively we resolve a conflict depends more on our emotional skills and emotional management than our opinions or viewpoints (Ortega Ruiz, 2010). Furthermore, some settings, such as school, can lead to greater sensitivity to conflicts in some individuals, who may need additional training to cope with the different emotional states they experience (Quiñones-Camacho and Davis, 2019). When such training is provided and the strategies are adequately implemented, conflicts can be opportunities for people to learn about new resolution strategies and responses and, thus, can ultimately facilitate their social development (Villamediana et al., 2015).

This paper aims to present a theoretical model covering emotion regulation (ER) and assertive conflict resolution. After gathering the existing evidence so far and taking into account the previous findings, the model was developed and used as the theoretical framework for the development of two interactive software programs: Happy 8–12, for children aged 8–12 years, and Happy 12–16, targeting adolescents aged 12–16 years. Traditional schooling is, many times, viewed as boring for students not showing the best results (Dicheva et al., 2015). Because of this and following the latest tendency found in research, the program was designed as a videogame in order to ensure the engagement and motivation of the students and learn the process to solve conflicts, as well as a perceived self-confidence and freedom to act in the way they considered best. An interactive approach to the program offers, as well, the possibility to include game elements, rewards, leaderboards, points, narrative, avatars, and role-play, as it has been shown to be effective in education (Li et al., 2022). It was, thus, hypothesized that Happy interactive programs would help to improve the management of emotions of students and also, at the same time, would help academic performance, climate center, and psychological wellbeing. Both programs have been implemented in Spain with positive initial results, which will also be discussed below. After obtaining positive results from the programs, which follow the aforementioned theoretical program, we aim at presenting this theoretical framework in the present study.

Building on the latest findings on emotion management models, such as Gross’s model (Gross, 2007, Gross’, 2015), social skills, and conflict resolution, the present theoretical model attempts to define a set of parameters and steps to enhance the learning of emotional skills from very early ages with the aid of the two interactive programs that were mentioned above. Training in these skills is expected to be transferrable to the management of real-life conflict situations and, thus, enhance social and individual wellbeing.

This *zeitgeist* has arisen in the wake of recent research reflecting a growing interest in emotional variables and student wellbeing (Filella et al., 2016; Pena Garrido et al., 2016; Rueda and Filella, 2016; Rueda Carcelén et al., 2016). At the same time, increasing rates of violent episodes in educational contexts have raised awareness of the need to improve social dynamics among peers and other individuals (Cabello Cuenca, 2019). To this end, several emotional education programs have been developed and have become essential to fostering and improving emotional competencies. These proposals have also had a positive impact on school environments and on academic performance, among other variables that are directly or indirectly involved (Cabello Cuenca, 2019). Consequently, the skills developed and learned in evidence-based emotional programs are expected to be transferrable to young children and adolescents, improving their ability to manage conflict situations and enhancing their social skills, individual wellbeing, and other indirectly involved variables, such as academic achievement, above and beyond any biological or dispositional factors (Rueda Carcelén et al., 2016; McRae et al., 2017).

### The importance of emotions in conflict resolution

The theoretical framework presented here is premised on the idea that emotions play a fundamental role in anyone’s life, starting in childhood and continuing in adolescence until adulthood. These emotions can modulate both cognition—skewing or neutralizing it—and behavior, especially when the emotional intensity is very high (Redorta et al., 2006; Koole, 2009; Gross and Jazaieri, 2014). Emotions can become problematic when they occur in an inappropriate context or are too intense, too frequent, or last for too long (Werner and Gross, 2010; Gross and Jazaieri, 2014; Gross’, 2015). Research also shows that impaired ER strategies are strongly associated with dysfunctional behaviors and disorders (e.g., Vanderlind et al., 2021).

At the same time, theoretical frameworks, such as the one presented here, must follow a global approach that considers different variables, including, as was mentioned in the previous section, social variables and the neurological bases of emotions. It is important to remember that the concept of emotions also includes the emotional brain and, more specifically, the limbic system, which is closely related to our emotions and memories. There is a scientific consensus that the amygdala is a critical region for generating, expressing, and experiencing emotions. The amygdala is studied as a whole, even though network interactions are quite complex (LeDoux, 2007).

LeDoux (1999) was one of the first authors to explain the role of emotions at a neuroscientific level. Numerous empirical papers have validated his findings, enabling an understanding of one of the most widely studied ER strategies, namely, *avoiding reactivity*. This strategy, closely linked to impulsivity, involves delaying a response by counting to 10 and stopping to breathe before responding to an intense and/or conflictive emotional situation. Subsequent studies have provided further evidence for these findings and enabled a more specific understanding of the structures involved in ER, including the prefrontal cortex and the brain’s association areas, which further interact with personality and temperament features (Morawetz et al., 2017).

Altogether, ER skills play a key role in adaptive behavior by enabling better social adaptation and productivity (Barreiros et al., 2019). The generation of negative emotions triggers ER processes to modulate responses to them (Davidson, 1998). ER is understood as individual attempts to adjust emotions as they appear and the way they are experienced or expressed. This process can be automatic, controlled, conscious, or unconscious (Carthy et al., 2010).

Consequently, emotions and their management determine our behavior, modulate our experience, and cause physiological changes that prepare us to take action (Gross, 2002, 2014; Ochsner and Gross, 2005). We cannot make decisions without taking emotions into account, and we cannot let our emotions take complete control (Bisquerra, 2009; Filella, 2014).

Understanding and training these emotions is, thus, crucial to ensuring individual wellbeing and adaptation to the environment.

### Current findings in emotion regulation among children and adolescents

To fully understand the functioning of the aforementioned kind of programs, it is important to note that ER processes have some specific characteristics during childhood and adolescence, which are discussed below.

Although it is well known that managing emotions allows children and adolescents to inhibit non-adaptive impulses, to direct their behavior in a constructive manner, and to be better accepted by their peers (e.g., Eisenberg et al., 2001, 2003), the development of such skills is very much linked to the stages of development. In this sense, ER skills are very much related to neurobiological factors, as research has widely shown (Lewis et al., 2007; Thompson et al., 2008). To be more specific, different regulatory processes, including the prefrontal cortex and the limbic system, as was mentioned above, are activated simultaneously organizing themselves to integrate perceptions, evaluations, and coordinate aspects that will deal with a particular situation (Lewis et al., 2006). Thus, neurobiological factors differ among children of different ages, resulting in more efficiency as the children become older.

Environmental factors have proved to complement this maturation of the nervous system for ER processes, with research showing that warm emotional environments, lack of poverty, and warm communities play a big role in the managing of emotions (e.g., Kim and Cicchetti, 2010; Cui et al., 2014).

In any case, humans learn to regulate their emotions in gradual and continuous progress, taking into account the factors mentioned above. This process starts at birth, with an absolute dependence on caregivers for managing emotions and it reaches adulthood with individuals being more autonomous and mature in terms of emotional management (e.g., Cole et al., 2004; Gross and Thompson, 2007; Thompson and Goodman, 2010). In this sense, children aged 8–12 years tend to focus on emotional skills related to analyzing the environment and responding to environmental needs, while adolescents are generally more focused on how they are perceived by others and how accepted they are in a group (Sabatier et al., 2017).

### Classification and taxonomies of emotions underlying the present theoretical framework

Considering the above, it is important to ask how exactly emotions work and how they can be classified to enable their adaptive management. There are currently more than a thousand definitions of the concept of emotion (Reisenzein, 2007), as well as numerous theories and hypotheses concerning how emotions work. Emotion can be defined as a complex state of the organism characterized by a state of arousal or disturbance that predisposes it toward an organized response (Bisquerra, 2000). Thus, emotions are not static but generated in response to an event, which may be internal or external, conscious or unconscious, and real or imaginary. A person’s thought pattern will, thus, greatly influence his or her emotions and, by extension, his or her actions (Lindner, 2006; Bar-Tal et al., 2007).

There is a wide variety of emotion taxonomies and classifications (Ekman, 1972). The most recent categorizations agree on classifying emotions as positive, negative, or ambiguous (e.g., Lazarus, 2000; Bisquerra and Mateo, 2019) to determine which ones cause distress and how to regulate them.

### Handling emotions

Experts agree on the importance of managing and regulating emotions properly (Gross’, 2015). However, to regulate emotions, one must first be aware of both one’s own and those of others, as this is what allows a person to consider other peoples’ views in the event of a conflict and to look for solutions (Maiese, 2006).

#### Emotional awareness

Emotions inform us of how we are feeling and what we want to achieve. If we are not aware of them, we will not be able to regulate them properly (Thompson et al., 2009).

Thompson et al. (2009) describe two components of emotional awareness: emotional attention and emotional clarity. The first refers to how people notice, think about, and monitor their state of mind, while the second refers to the clarity needed to understand, discern, and label emotions. These authors show that emotional clarity is inversely related to emotional variability. It follows that the more intense an emotion is, the easier it will be to pay attention to it (Thompson et al., 2009). These two emotional skills are also included in the classic model by Mayer and Salovey (1997).

Emotional awareness allows people to focus their attention on themselves, on others, and on situations (Chen et al., 2009) to act consciously and voluntarily, considering how to achieve their goals (Reijntjes et al., 2007). People with a high level of emotional awareness are less likely to display biases related to their state of mind in their judgments (Ciarrochi et al., 2003).

#### Emotion regulation

As noted, ER refers to the ability to manage the emotions we experience, as they are experienced, as well as how we express them (Gross, 1998, 2002). During the ER process, people can increase, maintain, or reduce positive and negative emotions (Koole, 2009).

**FIGURE 1:**
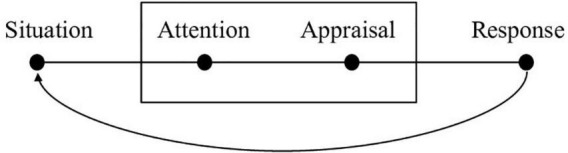
Basic process model of emotion regulation reproduced with permission from Gross and Barrett (2011, p. 12).

One of the ER models to achieve the greatest scientific recognition is Gross’s modal model of ER (Gross, 1998, Gross’, 2015). This model aims to explain the process whereby a given external or internal situation triggers an emotional response and is eventually appraised by the person experiencing it. The process culminates in a response that is in line with the ER process (refer to Figure 1).

In keeping with a revised version published by Gross’ (2015), the modal model starts when emotion is generated. Subsequently, a second-order appraisal is generated, which refers to ER *per se*. The second-order appraisal consists of three stages that are necessary to be able to regulate emotions properly: identification, in which the person generates the need to regulate their emotions; selection, where they select the most effective strategy or strategies; and implementation, in which they put these strategies into practice (Gross’ 2015; Ford and Gross, 2018; Eldesouky and Gross, 2019).

However, training and learning are needed to be able to select the optimal strategy for the emotion generated and the situation experienced at any given time.

## The conflict resolution model

The interpersonal conflict resolution model presented here is based on emotion management, as described above. It consists of the following four-step process.

### Step 1: Emotional awareness

As noted, emotional awareness can be understood as the ability to be aware of both our own and others’ emotions, to identify them with a proper and adequate vocabulary, and to be aware of the interaction between emotion, cognition, and behavior (Altarriba and Bauer, 2004; Damasio, 2004; Bisquerra, 2009; Thompson et al., 2009).

Once a person becomes aware of his or her emotions, the emotions experienced must be adequately legitimated. Tolerating and respecting others’ emotions will also help reduce anxiety in a situation of conflict (Redorta et al., 2006; Bisquerra, 2009; Filella, 2014). It is, thus, safe to say that the intensity and duration of an emotion depend on the following factors: Biology (DNA) + Environment (LIFE) + Situation.

Emotional awareness is, thus, achieved when the emotion becomes a conscious feeling (Mayer and Salovey, 1997). The importance of prevention lies in the demonstrated relationship between good emotional awareness and lower rates of depression and mental disorders (Conner et al., 2019; Huggins et al., 2021).

### Step 2: Avoiding reactivity

Once a person has become aware of their feelings and legitimated their emotions, they must reduce the intensity thereof by avoiding reactivity and impulsivity and increasing their positive emotions. Otherwise, it will be more difficult for them to regulate their emotions properly (Schreiber et al., 2012; Gross, 2014), which could increase the likelihood of cognitive problems or emotional disorders, such as phobias, stress, or depression (Bisquerra, 2009; Werner and Gross, 2010; Gross, 2014).

Impulsiveness negatively affects the ability to concentrate on meaningful thoughts, planning actions, and objectives (Wittmann and Paulus, 2009). It can also affect cognitive reasoning skills (Schreiber et al., 2012).

To date, the most widespread strategies and techniques to improve emotional awareness and reduce reactivity are relaxation and breathing techniques (Bisquerra, 2009; Ruiz et al., 2012). The autonomic nervous system has been shown to need from 20 min to several hours to return to a state of calm (Sapolsky, 2008). Therefore, the “traffic light technique” (Goleman, 1995) succeeds in reducing emotional intensity. It consists of visualizing a traffic light that begins by turning red (encouraging the person to stop), then yellow (breath), and finally green (think and act).

### Step 3: Emotion regulation strategies

This third and most decisive step incorporates the strategies for handling emotions from Gross’s ER model (2007), as well as strategies focused on antecedents, thereby combining distraction and reappraisal strategies.

#### Strategies for handling emotions

##### Expressing emotions

When we experience a significant, emotionally impactful event, we feel a need to share it with family (Zech and Rimé, 2005) or find other ways to express it. Expressing emotion allows us to focus on new events and leave old ones behind. If these memories do not become latent and remain in our awareness for too long, they may cause certain negative effects. This may happen because of the presence of intrusive thoughts and images, which lead to rumination, which, in turn, can negatively affect emotional recovery (Rimé, 2009).

##### Verbal emotional expression

Widely used in psychotherapy, verbal emotional expression forces the person to turn emotions into feelings, thereby fostering emotional awareness and understanding. The person, thus, perceives an emotional, cognitive, and interpersonal benefit, which contributes to his or her emotional recovery (Zech and Rimé, 2005; Rimé, 2009).

##### Written emotional expression

There are situations in which people cannot communicate verbally with others, either because they do not know how to do so properly or because expressing their emotions is culturally frowned upon (Butler et al., 2007; Lu and Stanton, 2010).

Two types of interventions involve working on written emotional expression: positive writing (Wing et al., 2006; Suhr et al., 2017) and writing about traumatic, meaningful, or stressful events (Baikie and Wilhelm, 2005; Gortner et al., 2006; Lu and Stanton, 2010).

When these methods are used for written expression, it increases both satisfaction with life and emotional intelligence (Wing et al., 2006), including in the long term.

Additionally, it reduces physical symptoms (Lu and Stanton, 2010) and visits to health centers and sick leave (Baikie and Wilhelm, 2005), mitigates the decrease in positive affect caused by the event (Baikie and Wilhelm, 2005; Lu and Stanton, 2010), reduce symptoms of depression (Gortner et al., 2006), and increases feelings of wellbeing (Baikie and Wilhelm, 2005).

#### Strategies focused on antecedents

##### Distraction

Distraction is used as a method of disconnecting from negative emotions, acting as a filter that blocks emotional information before it can be appraised; it is very effective in modulating high-intensity emotional stimuli (Sheppes et al., 2011, 2014). Ceasing to think about a negative situation or emotion helps prevent rumination, an activity that can sustain a negative emotional state (Augustine and Hemenover, 2009). Meta-analyses (e.g., Webb et al., 2012) also show that distraction has a positive and significant effect on ER.

A distinction can be drawn between behavioral distraction strategies (e.g., engaging in an activity other than the one causing concern) and cognitive distraction strategies (e.g., thinking of things other than the issue that concerns us or is generating negative emotions), although both types have very similar results in terms of ER.

##### Reappraising the situation

The most difficult and effective strategy aims to change to a more positive way of thinking and refocus the conflict from another point of view to find a solution. It is effective at the affective, cognitive, and social levels.

The affective consequences of reappraisal include a lessening of negative experiences and emotional expression and an increase in positive experiences and emotional expression (John and Gross, 2004; Ochsner and Gross, 2005; Augustine and Hemenover, 2009; Gyurak et al., 2011; Gross, 2014). Although it does not have observable physical consequences (Gross, 2002), it can potentially decrease physiological activity (Gross and John, 2003; Ochsner and Gross, 2005; Gyurak et al., 2011; Gross, 2014). Using this strategy may help a person restore their state of mind (John and Gross, 2004).

As for the cognitive benefits of reappraisal, first, it does not require a constant effort of self-regulation, as it is used at the beginning of the process of generating emotion. Additionally, unlike suppression, it does not affect memory (Gross, 2002, 2014; John and Gross, 2004; Ochsner and Gross, 2005). Finally, reappraisal has been shown to successfully modulate low-intensity emotions and allow emotional processing (Sheppes et al., 2011).

At the social level, better interpersonal functioning increases the likelihood of being loved by others and boosts personal wellbeing (Gross, 2002, 2014; Gross and John, 2003; John and Gross, 2004; Gyurak et al., 2011).

People who frequently use this strategy are characterized by having higher self-esteem and an optimistic attitude toward stressful situations, reinterpreting them, and actively seeking to repair the negative states of mind that they create in them; they are also able to express their positive and negative emotions socially (John and Gross, 2004).

##### Looking for a solution

Although regulation strategies are effective, if there is a conflictive event that creates anxiety or distress, a solution must be sought. In fact, in conflicts for which there is a solution, tools need to be provided to facilitate this resolution. A good example might be Krumboltz’s (1979) traditional decision-making model, whose steps are as follows: (1) define the problem; (2) establish a plan; (3) identify alternatives; (4) self-appraisal; (5) investigate possible results; (6) eliminate low-benefit, high-cost alternatives; and (7) experiment.

When the conflict exceeds one’s capabilities, it is important to consider the possibility of *asking/looking for help*, i.e., explaining the situation to someone with authority, such as a mediator, teacher, or parent. This is a useful strategy in complex conflict situations (mobbing, bullying, etc.).

It is also crucial for students to put these different strategies into practice. As students need to deal with and overcome emotional and motivational challenges when undertaking various types of academic tasks, having a wide range of emotional strategies can help them overcome motivation affectation and promote wellbeing (Alonso-Tapia et al., 2020).

### Step 4: Assertive communication and behavior

Social skills are key to achieving a positive outcome after a conflict. These skills include proper verbal and non-verbal communication between the parties in conflict and empathy. Akamatsu and Gherghel (2021) underline the role of empathy in preventing manipulation and antisocial behaviors.

In the last few decades, various authors have put forward models to define how people act when faced with a conflict. For example, Caballo (1997) proposed a two-dimensional model characterized by the person’s expression (manifest or hidden) and way of expressing themselves (whether they invoke punishment or threats). Using this combination, the model identifies four types of behavior, which have subsequently been collated and defined in more detail by other authors (Roca, 2014):

1. Passive behavior: In these situations, the person neither expresses him or herself nor displays behaviors of punishment or threat. This means that there is no expression of thoughts, feelings, or wishes.
2. Aggressive behavior: In these situations, the person does not respect the rights, desires, or feelings of others. Conflicts are solved through imposition and authoritarianism.
3. Passive-aggressive behavior: Of the aforementioned authors, only Caballo (1997) takes this type of behavior (*negativistic behavior*) into account. In this case, the person does not express himself or herself and displays the behavior of punishment or threat.
4. Assertive behavior: In this situation, people communicate by adequately expressing their feelings, thoughts, and interests, while also respecting those of others without hurting them. This type of behavior facilitates conflict resolution by fostering appropriate communication and respect among the parties to the conflict.

**FIGURE 2:**
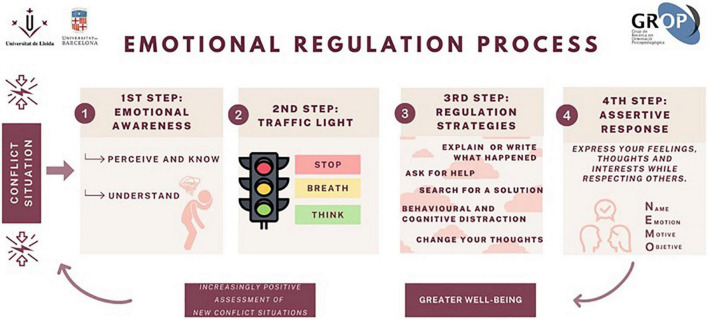
Steps followed in the Happy software’s following the emotional regulation process (GROP). All participants have to follow these steps when facing a conflict during the game. If the process is successfully completed, the participant will get a high score. Contrary to that, if the participant obtains a low score, the need of training the process will arise (Reproduced with permission from Guiu et al., 2007).

To encourage assertive communication, the emotion management model proposed here works using positive communication. NEMO is a technique for implementing this in everyday life. It stands for: Name (name of the person being addressed) + Emotion (the emotion they have generated in the speaker) + Motive (what caused the emotion to be generated) + Objective (what the speaker aims to achieve by communicating with them) (see Figure 2).

## The Happy 8–12 and Happy 12–16 interactive programs

The abovementioned theoretical framework served as the basis for the Happy 8–12 and Happy 12–16 emotional education intervention programs. Both include specialized software for working on emotional skills focused on conflict resolution by means of the theoretical model presented in this article, targeted at children aged 8–12 years and adolescents aged 12–16 years, respectively. According to the literature, there is a promising direction in prevalent technologies, such as videogames and wearables, that indicates that emotion and social situations can be more optimally represented in this kind of training (Sadka and Antle, 2022).

Taking this into consideration, Happy software was created and designed with the idea of increasing the motivation of students and ensuring engagement with the training programs. All the content is presented in the form of a video game, as today’s children and adolescents have been surrounded by and immersed in such technology since they were born. Similarly, communication technologies and “phygital” media have been shown to captivate, interest, and motivate these digital native generations, directly impacting their learning processes (D’Amico, 2018).

Both programs are video games designed specifically to help children and adolescents learn to manage their emotional skills better and, thus, be able to respond assertively and with greater associated wellbeing to conflicts arising in their everyday life. In order to achieve that, Happy videogames were designed in a way that students can feel identified with the situations they live daily in school: different situations are presented in the video game and students have to use their resources, knowledge, and emotional management in order to solve them in the most assertive way that is possible. When using the software, however, teachers and students also have a teaching guide, which is a part of the training program, to help them follow the virtual training (Rueda Carcelén, 2017).

The program should ideally be used in tutorial activities across all courses throughout the academic year, as the preliminary results obtained so far show (for example, Ros-Morente et al., 2018).

**FIGURE 3:**
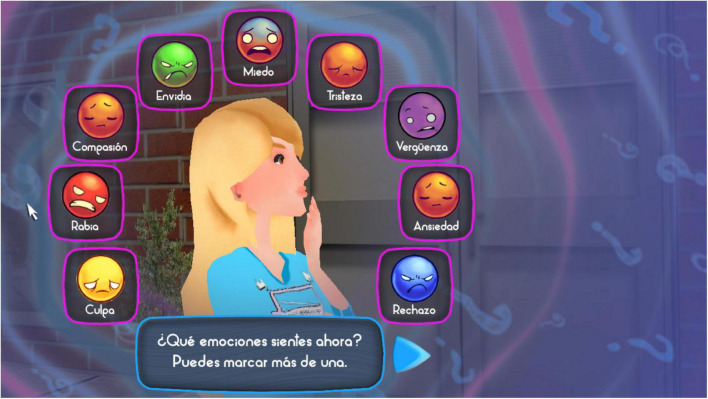
Image of the first step (Emotional awareness indicated in Figure 2) in the Happy 12–16 software.

In order to train students in emotional awareness, under the present conflict resolution model, the first step in the program is to become aware of the emotions one feels when a conflict is taking place. In this step, students are shown different drawings of a face representing different emotions (refer to Figure 3). To encourage their emotional awareness, they are asked to select one (or more) emotions that reflect what they are feeling. Each student has previously selected and defined an avatar, which can be a girl or a boy, to virtually represent them as a player. This approach is thought to help students identify and engage more with the educational game (Collado Temprado, 2015).

**FIGURE 4:**
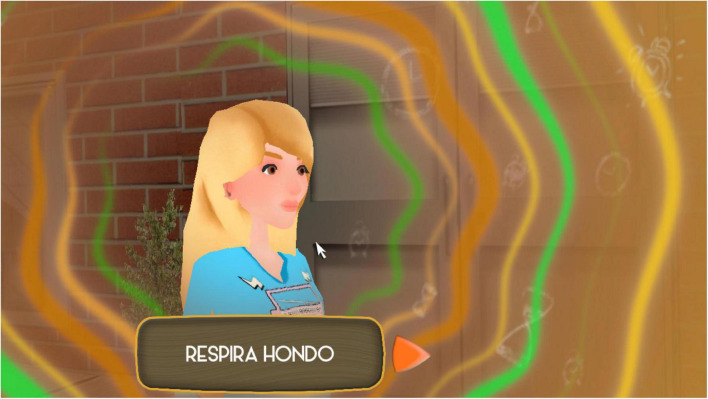
Image of the traffic light technique included in the educational game Happy 12–16 corresponding to the second step of the process (see Figure 2).

The second step involves the aforesaid traffic light technique (Goleman, 1995). Students are prompted to breathe deeply and avoid reactivity before continuing with the educational game (see Figure 4).

**FIGURE 5:**
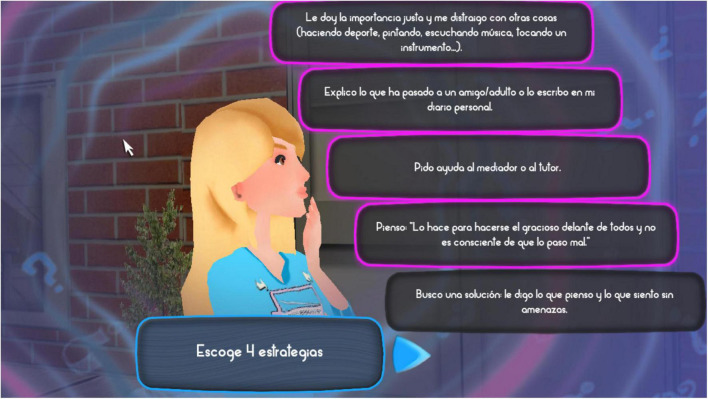
Image of the third step of the conflict resolution process in the software Happy 12–16, which shows the different regulation strategies to solve a conflict.

When students reach the third step, they are shown a range of various options and strategies, which they can choose from to solve the conflict situation they have been presented with. Some of the displayed ER strategies are more adaptive than others, as explained earlier. Players earn the highest scores by choosing the most efficient strategies, normally based on reappraisal. This training is expected to improve students’ choice of regulation strategy when confronted with a conflict situation (Figure 5).

**FIGURE 6:**
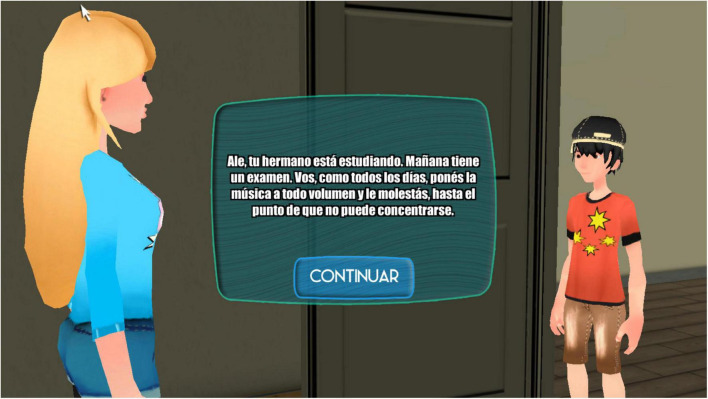
Image of the final step of the process (N + E + M + O strategy).

Finally, Step 4 corresponds to the NEMO technique described in the previous section, as shown in Figure 6.

Although both the Happy 8–12 and Happy 12–16 programs follow the same steps, they are adapted to their respective age groups. Both present players with 25 conflicts, 15 of which take place in a school environment (e.g., “Nuria is a girl who tells lies to win friends.”) and 10 between siblings in a family environment (e.g., “You are playing chess with your brother; when he loses, he accuses you of cheating.”). For each conflict, the student has to follow the process, choosing from different possible responses, with the most assertive one being the only right choice.

To date, several pilot studies have been conducted, which have been very well received by the educational community in Spain and have compared the results of those children who have received training with Happy software and those who have not. The initial results obtained so far suggest that the programs have positive effects.

Before starting the training with Happy software, the research was presented to the Education Department of Catalunya, obtaining official authorization for the project. After this first step, schools that were interested in trying the program were invited to participate. At the same time, all the scholars were invited to participate voluntarily and anonymously in the studies. Participation only took place after participants and their families were informed in depth about the study.

It is also important to mention that those teachers involved in the training received intensive training before the administration of the program. In this way, any kind of skewness in the training with Happy programs was avoided.

### Results of application of the program in primary school

The first program tested on a population of students was the primary education program, i.e., Happy 8–12, which was used at several primary schools in the city of Lleida (Spain) with a total sample of 576 students (Cabello, 2018; Cabello Cuenca et al., 2019).

A specific protocol, combining qualitative and quantitative measures, designed for the study was applied at pre- and post-assessment moments. Regarding qualitative measures, opinions on the program of teachers and students were gathered, showing the high satisfaction and motivation of both groups. Quantitative measures included different questionnaires such as the Emotional Development Questionnaire (QDE; Pérez-Escoda et al., 2021); Evaluation of Self-esteem in Elementary Education (A-EP; Ramos et al., 2006) for emotional competencies; anxiety-trait was assessed with State-Trait Anxiety Inventory for Children-Spanish Version (STAIC; Spielberger et al., 1982); Social Classroom Climate Questionnaire (CES; Trickett and Moos, 1974; Pérez et al., 2010) was administrated for social climate in the class; Climate Playground Questionnaire (CPQ; Developed *ad hoc*) tapped the climate in the playground, and, finally, academic performance was gathered with the marks of the compulsory subjects of the students.

Quantitative data were analyzed with SPSS 27 software. Different analyses were carried out, including correlations among variables, regression, and implementation of the General Linear Model (GLM) for repeated measures. The results obtained and published in different articles (Filella et al., 2016; Cabello, 2018; Cabello Cuenca et al., 2019; Ros-Morente et al., 2019; Filella Guiu, 2022, among others) after a pre-post design with a control group showed that training with the Happy 8–12 software for an academic year significantly improves emotional skills and reduces students’ levels of anxiety (*p* < 0.001 in both cases), improving at the same time other variables. Although the effect of this learning was discreet, it should be recalled that these variables—emotional skills and the social climate of the classroom—require an extended time and training to change completely and will not show definitive progress until more training is implemented.

### Results of the use of the program in secondary schools

As with the primary school students, a pilot study with a pre-post design was conducted. Similar instruments were administered for the evaluation protocol, although fewer variables were assessed, in order to have more focus on emotional competencies during adolescence. These instruments, although assessing the same variables as in the primary school students sample, were adjusted to the age of the population (adolescents). Also, in order to evaluate the effects of the program, a protocol combining qualitative and quantitative measures was administered. Specifically, qualitative results were gathered by asking teachers and students about their opinions on the training and the program. Quantitative data were collected by self-administered questionnaires. Concretely, the instruments that were administered to students were the following: for emotional competencies, the Emotional Development Questionnaire for secondary school (QDE SEC; Escoda, 2016) was administered. Anxiety-state was assessed with the Spanish version of the State-Trait Anxiety Inventory (STAI; Spielberger et al., 1982). Finally, academic achievement was evaluated with the average marks of all the subjects that secondary students undergo in secondary school, that is, Biology and Geology, Geography and History, Spanish Literature, English Literature, Physical Education, Ethics, and three different optative subjects.

Quantitative data were analyzed with SPSS 27 software. Data results were studied with analysis including correlations among variables, regression, and General Linear Model (GLM) for repeated measures, as was also the case in the primary education population. The results of the study with secondary school students (*n* = 903), published in different articles (e.g., Rueda et al., 2015; Filella et al., 2018; Cabello Cuenca et al., 2019, among others) show that individuals who received training were more likely to improve their emotional competencies (*p* < 0.001). Also as with the primary school students, the effect of the learning process on emotional skills was modest and the effect sizes were small for all the scales except anxiety (0.2–0.4).

It is worth noting that emotional awareness seemed to have a more striking effect in the sample of adolescents. Far from being surprising, this effect is understandable, as it clearly reflects the natural acquisition of emotional competencies and training in them, which first requires becoming aware of one’s emotions (refer to Figure 1; Gross and John, 2003). Additionally, prior research has shown that similar constructs, such as lower emotional clarity, may be related to specific ER strategies, such as suppression, indicating that higher emotional awareness may be beneficial for choosing more adaptive strategies (Arndt et al., 2018).

In both samples, the trend toward improvement in academic achievement variables was significantly different in the experimental group, with a medium effect size. Although previous studies point to the importance of social and emotional levels for improving academic achievement (Dotterer and Lowe, 2011), these findings can probably be explained by the fact that the grades were given right after the training with the software was completed, at the end of the academic year. In this sense, a long-term reassessment might yield more detailed results.

### Conclusion on the implementation of Happy programs

After the development of the final theoretical framework and the first pilot studies with Spanish populations research show that, as was expected, those individuals who received training in schools or high schools with the Happy interactive program exhibited a higher and significant tendency to improve their emotional competencies. Differently, when analyzing the changes in the control group, there were no important differences in their levels of emotional competencies.

It is safe to state, however, that the effect of the learning process on emotional skills was significant at a statistical level, but the effects seem modest. This is congruent with the idea that variables, such as emotional competencies, especially ER, involve a certain amount of stability and require a long time for their total change and training since important cognitive structures are involved.

Also, these first studies hint at the idea of how important teacher’s communities and families are, in order to ensure the efficacy of the program. For this reason, it is highly important to take into consideration the involvement of families and its correlates in future studies.

In conclusion, Happy software is conceived as an opportunity to develop and improve emotional competencies in children. However, future research should continue to assess this model’s effects, especially in the mid-to-long term, to prove its efficiency and efficacy. Furthermore, additional research should be conducted on the training process to learn more about how the various competencies involved in conflict resolution are acquired.

Finally, studies carried out so far have shown that more training for teachers and families may be helpful. Despite the positive results of the software, these are not independent of the environment or the motivation and implication of professionals. Therefore, future studies should also focus on a higher implication of families and professionals during the training.

## Discussion

During the latest decades, interest in ER and emotion management in education has exponentially increased (Linnenbrink, 2006; Jacobs and Gross, 2014, for instance). Proper management of emotions has been linked to many correlates, such as better academic achievement (Dotterer and Lowe, 2011; Rueda Carcelén et al., 2016; McRae et al., 2017), increased wellbeing and decreased anxiety (Bisquerra, 2009; Filella, 2014), less conflictive behavior (McRae et al., 2017), and better school adjustment (Leppänen and Hietanen, 2001), just to mention a few.

Evidence shows, thus, that training emotional management or, similarly, emotional competencies, can be very beneficial for students in order to improve variables related to academic performance, but also to wellbeing and prevention of conflicts or even bullying (Smith and Low, 2013; Filella Guiu, 2022, among many others).

Programs and interventions regarding this background have emerged with great success during the last years (e.g., Graczyk et al., 2000). In addition, interactive technologies seem to offer several advantages when learning in schools, such as an increased level of motivation among the students, more self-confidence, and a higher engagement in the training (Dicheva et al., 2015).

In the present article, we tried to thoroughly explain the theoretical framework from two interactive programs in the format of a video game (Happy 8–12, for children aged 8–12, and Happy 12–16, for adolescents) which was developed during years of experience and evaluation in the University of Lleida, Spain (Cabello, 2018; Filella et al., 2018; Ros-Morente et al., 2019; Filella Guiu, 2022, among others) with positive results.

Happy software is designed for learning how to manage emotional competencies properly, in both adolescence and childhood, and, thus, improve other variables such as wellbeing, classroom climate, or academic achievement and they are fundamental in the theory that is described in the present study. This framework is based on the evolutive theories, taking into account the pentagonal model of competencies of Bisquerra (2000) and Gross’ (2015), and it takes into account the Piaget-Kohlberg moral stages, along with the theoretical findings of the latest decades.

Henceforth, as it has been explained, results obtained from studies carried out in different schools and with a total of more than 3,000 students show promising results.

Taking all this into account, the authors aimed at presenting the theoretical framework in which the software and interventions are framed, in order to set the bases for future investigations and software developments.

## Data availability statement

The original contributions presented in this study are included in the article/supplementary material, further inquiries can be directed to the corresponding author.

## Author contributions

GF developed the idea for Happy Software and contributed to the entirety of this manuscript. AR-M participated in all phases of the present research and the implementation of the interactive programs. Both authors listed have made a substantial, direct, and intellectual contribution to the study, and approved it for publication.
